# Effect of the saliva from different triatomine species on the biology and immunity of TLR-4 ligand and *Trypanosoma cruzi*-stimulated dendritic cells

**DOI:** 10.1186/s13071-016-1890-x

**Published:** 2016-12-09

**Authors:** Maria Tays Mendes, Tamires Marielem Carvalho-Costa, Marcos Vinicius da Silva, Ana Carolina Borella Marfil Anhê, Rafaela Mano Guimarães, Thiago Alvares da Costa, Luis Eduardo Ramirez, Virmondes Rodrigues, Carlo Jose Freire Oliveira

**Affiliations:** 1 Department of Biological Sciences, University of Texas at El Paso, El Paso, TX USA; 2 Laboratory of Immunology, Federal University of Triângulo Mineiro, Uberaba, Minas Gerais Brazil; 3 Undergraduate Course of Environmental Engineering, Federal University of Triângulo Mineiro, Uberaba, Minas Gerais Brazil

**Keywords:** Triatomines, Saliva, Dendritic cells, *Trypanosoma cruzi*, Immunomodulation

## Abstract

**Background:**

Triatomines are blood-sucking vectors of *Trypanosoma cruzi*, the causative agent of Chagas disease. During feeding, triatomines surpass the skin host response through biomolecules present in their saliva. Dendritic cells (DCs) play a crucial role in the induction of the protection to aggressive agents, including blood-sucking arthropods. Here, we evaluated if salivary components of triatomines from different genera evade the host immunity by modulating the biology and the function of LPS- or *T. cruzi*-stimulated DCs.

**Methods:**

Saliva of *Panstrongylus lignarius*, *Meccus pallidipennis*, *Triatoma lecticularia* and *Rhodnius prolixus* were obtained by dissection of salivary glands and the DCs were obtained from the differentiation of mouse bone marrow precursors.

**Results:**

The differentiation of DCs was inhibited by saliva of all species tested. Saliva differentially inhibited the expression of MHC-II, CD40, CD80 and CD86 in LPS-matured DCs. Except for the saliva of *R. prolixus*, which induced IL-6 cytokine production, TNF-α, IL-12 and IL-6 were inhibited by the saliva of the other three tested species and IL-10 was increased in all of them. Saliva per se, also induced the production of IL-12, IL-6 and IL-10. Only the saliva of *R. prolixus* induced DCs apoptosis. The presence of PGE_2_ was not detected in the saliva of the four triatomines studied. Finally, *T. cruzi* invasion on DCs is enhanced by the presence of the triatomine saliva.

**Conclusions:**

These results demonstrate that saliva from different triatomine species exhibit immunomodulatory effects on LPS and *T. cruzi*-stimulated DCs. These effects could be related to hematophagy and transmission of *T. cruzi* during feeding.

**Electronic supplementary material:**

The online version of this article (doi:10.1186/s13071-016-1890-x) contains supplementary material, which is available to authorized users.

## Background

During feeding, triatomines and other arthropod vectors trigger a series of events leading to modulation of immune and hemostatic responses of their hosts. For this purpose, the saliva of these arthropods is released directly into the blood vessels and the surrounding tissues [1, 2] and contains an arsenal of molecules with anti-hemostatic and immune-modulatory activities [3, 4]. Dendritic cells (DCs), present in different tissues including the skin, comprise a population of highly specialized white blood cells that recognize, capture, process and present antigens [5, 6]. The antigen presentation capacity by these cells is an important step in the stimulation of T lymphocytes against various harmful agents including bloodsucking arthropods and the pathogens they transmit [7, 8].

With regard to the presence of triatomine salivary molecules that antagonize the host hemostatic system, several studies have already shown the presence of substances with vasoconstrictive, anti-platelet and anti-coagulant properties [3, 4]. In the case of immunomodulatory components, few molecules have been characterized so far. In this particular case, it has been shown that *Rhodnius prolixus* is capable of producing a class of histamine-binding proteins called nitrophorins. This seems to be very important during blood-feeding, since the release of histamine by basophils and mast cells can induce inflammatory reaction with increased vascular permeability, plasma exudation and itching induction in the bite site [9–11]. More recently it has been shown that *R. prolixus* can secrete lysophosphatidylcholine (LPC), a lipid molecule present in saliva that may play a role in modulating macrophage and facilitating the transmission of *T. cruzi* to their hosts [12, 13].

Although there are few studies, we believe that the success of the infestation, feeding and transmission of pathogens by triatomines involves the modulation of many other key components of the immune system, such as DCs. Moreover, is likely that the number of molecules with potential properties in triatomine saliva may be much higher than described so far. This fact can be easily confirmed in salivary transcriptomes and proteomes (sialomes) [14–16] or the complete genome description of *R. prolixus*, with many previously unidentified molecules with probable role in modulation of the immune system [17]. Thus, it increasingly stimulates our search for molecules and bioactive properties present in the saliva of these insects that, despite their medical importance, have been studied little so far with respect to their immunomodulatory capacity on hosts.

In summary, although data in the literature showing the modulatory effects of triatomine saliva on the hemostatic system exist, studies evaluating the effect of the saliva on cells of the immune system are still incipient. Thus, the purpose of this work was to investigate the effect of saliva of four triatomine species, *Panstrongylus lignarius* (also known as *Panstrongylus herreri*), *Meccus pallidipennis*, *Triatoma lecticularia* and *R. prolixus*, on the biology and function of DCs, important cells of the immune response against hematophagous arthropods.

## Methods

### Triatomines

Four triatomine species were used (*P. lignarius*, *M. pallidipennis*, *T. lecticularia* and *R. prolixus*) from colonies of Parasitology Department’s insectary, Federal University of Triângulo Mineiro - UFTM, Uberaba-MG, Brazil. The insects, independent of the life-cycle, were fed using chickens as blood source.

### Saliva collection

Saliva from adult triatomines was obtained as described by Mesquita et al. [12]. Triatomines of the four species, from both sexes, and after different days of fasting (7 to 21 days) were fixed on ice and thereafter cleaned with water and ethanol 70%. With forceps, the triatomines *R. prolixus* had their heads pulled, allowing exposure of the salivary glands and their collection. To collect the glands of other species, the side of the abdomen and chest were cut up and after opening and exposure of the thoracic contents, under stereomicroscope, the glands were located and collected. Glands were kept on ice throughout the procedure, and every three pairs of glands (three insects) were added to 10 μl of sterile saline. The pool of saliva from each species was obtained from glands collected 7 to 21 days post-feeding. With the aid of sterile needles, these salivary glands were pierced to allow leakage of saliva and centrifuged at 11,000× *g* for 5 min. The supernatant collected was kept at -70 °C until it was used. The protein concentration was determined on a pool of saliva by the Bradford method (Pierce, Rockford, IL, USA) and the values obtained were *c*.26 mg/ml for *P. lignarius*, *c*.29 mg/ml for *M. pallidipennis*, *c*.25 mg/ml for *T. lecticularia*, and *c*.20 mg/ml for *R. prolixus*.

### *Trypanosoma cruzi* strain

The blood trypomastigote forms of the *T. cruzi* (Y strain) were obtained after infection of green-monkey kidney epithelial cells (LLC-MK_2_) incubated at 37 °C in a humidified atmosphere containing 5% CO_2_ in DMEM supplemented with 10% fetal bovine serum (Sigma, St. Louis, MO, USA).

### Animals

C57BL/6 (6–8 weeks old) wild-type mice were bred and maintained, as determined by the Ethics Committee on Animal Use (CEUA), in experimental animal facilities of the Federal University of Triângulo Mineiro - UFTM, Uberaba-MG, Brazil.

### Reagents

Recombinant murine Granulocyte-macrophage colony-stimulating factor (GM-CSF) was purchased from PeproTech (Rocky Hill, NJ, USA). Ultrapure lipopolysaccharid (LPS) from *Escherichia coli* 0111: B4 was purchased from Invivogen (San Diego, CA, USA). The doses of both molecules were determined based on the recommendations of the manufacturer and/or through our concentration-response studies. Cytokines kits (OptEIA™ ELISA), and antibodies were purchased from eBioscience (San Diego, CA, USA) or BD Biosciences (San Jose, CA, USA).

### Dendritic cells

DCs were obtained by differentiation of the cells of the bone marrow (BM) of mice as described previously by Oliveira et al. [18], with some modifications. Briefly, bone marrow from femurs and tibiae removed from C57BL/6 mice were cultured in 10 ml RPMI-1640 (Gibco, Grand Island, NY, USA) supplemented with 10% v/v inactivated fetal bovine serum (Gibco), 50 mM 2-mercaptoethanol (Sigma-Aldrich, St. Louis, MO), 1 mM sodium pyruvate (Sigma-Aldrich), 25 mM sodium bicarbonate (Gibco), 10 mM HEPES (Sigma-Aldrich), 100 UI/ml penicillin (Sigma-Aldrich), 100 μg/ml streptomycin (Sigma-Aldrich), 25 mM L-glutamine (Gibco), and murine GM-CSF (25 ng/ml). were obtained from Sigma-Aldrich (St. Louis, MO). Cells suspensions were prepared at 2.0 × 10^6^ cells/ml in Petri dishes. On the fourth day of culture, 10 ml of culture medium supplemented with GM-CSF (50 ng/ml) was added to the plate. After 7 days of culture, the cells were collected and analyzed by flow cytometry to determine the percentage of CD11b, and CD11c, and experiments were started only after evaluating this percentage.

### Evaluation of the effect of saliva on the differentiation of DCs

BM cells at a concentration of 2 × 10^5^ cells per well (48-well plate) in an initial volume of 200 μl of RPMI 10% with GM-CSF (25 ng/ml) were treated with different concentrations of saliva (dilution 1:30, 1:100, 1:300 and 1:1000, v/v) from different species on day 0 and 3 of culture. On day 4, 200 μl of RPMI 10% with GM-CSF (50 ng/ml) were added. Cells were assessed on the seventh day by flow cytometry for expression of CD11c, CD11b, MHC-II, CD40 and CD86.

### Evaluation of the effect of saliva on the expression of CD11b and CD11c in differentiated DCs

DCs on the seventh day of cell differentiation, at a concentration of 1.5 × 10^5^ cells per well (96-well plate) in a volume 200 μl of 10% RPMI were incubated for 18 h with different concentrations of saliva from the four triatomines and then assessed by flow cytometry for expression of CD11b and CD11c.

### Evaluation of the effect of saliva on the maturation of DCs

To evaluate the influence of saliva on maturation DCs, differentiated cells at a concentration of 1.5 × 10^5^ cells per well (96-well plate) in a volume of 200 μl of RPMI 10% were stimulated with different concentrations of saliva for 1 h and then with Toll-Like receptor (TLR)-4 ligand [lipopolysaccharide - LPS (100 ng/ml)] for further 18 h. The cells then were analyzed by flow cytometry for expression of stimulatory and co-stimulatory molecules, MHC-II, CD40, CD80 and CD86. Supernatants from these cultures were collected and levels of cytokines IL-12p40, IL-6, IL-10, and TNF-α were measured by ELISA.

### Apoptosis

DCs at a concentration of 1.5 × 10^5^ cells per well (96-well plate) in a volume of 100 μl RPMI 10%, were incubated with different concentrations of saliva from different triatomines for 18 h. After this time, the cells were removed from the plate, washed twice with PBS, and centrifuged at 400× *g* at 4 °C for 10 min. Then, the cells were resuspended in Annexin buffer (BD Pharmigem, San Jose, CA) and incubated for 15 min. After this time, Annexin-V conjugated to FITC (fluorescein isothiocyanate) and propidium iodide were added. Afterwards, cells were acquired on FACSCalibur flow cytometer and analyzed using CellQuest 5.1 software (BD Pharmigem) and FlowJo software (TreeStar Inc., Ashland, OR, USA). Viable cells were deemed negative for staining with Annexin-V andpropidium iodide. As a positive control for cell viability, DCs cultured in the absence of saliva were used and as positive control of cell death, cells maintained for 30 min at 57 °C were used.

### Flow cytometry analysis

The cultured cells were analyzed by flow cytometry according to the protocol described by Oliveira et al. [18]. The monoclonal antibodies used were: anti-CD11c, anti-CD11b, anti-MHC-II, anti-CD40, anti-CD80 or anti-CD86 labeled with APC, FITC, PE or PECy7 according to the intended purpose. The acquisition was made in a FACSCalibur flow cytometer (BD Immunocytometry Systems) and the analyzes through the CellQuest 5.1 and FlowJo software.

### Cytokine assays

The interleukin (IL)-12p40, TNF-α, IL-10 and IL-6 were measured by specific commercial kits, BD OptEIA (BD Biosciences) by ELISA type immunoassay “sandwich”. For the samples of IL-6 and IL-12p40, dilutions of 10 and 20 times were carried out, respectively. The concentration of the samples was estimated by comparing the absorbance obtained from the standard curve got by serial dilution of recombinant murine cytokines. Results were analyzed by linear regression with the aid of StatView program, and expressed in ng/ml or pg/ml.

### Prostaglandin concentration determination

The presence of prostaglandin-E_2_ (PGE-_2_) in the saliva of the species was evaluated by PGE_2_ EIA Kit (Enzo Life Sciences, Farmingdale, NY) according to manufacturer’s instructions. The analysis was performed at an absorbance of 405 nm with correction between 570 and 590 nm in a spectrophotometer. The PGE-_2_ concentration was determined by comparison with a standard curve. The detection limit for this assay was 13.5 pg/ml.

### *Trypanosoma cruzi* invasion assay

The *T. cruzi* invasion assay was done as described previously by da Costa et al. [19]. The trypomastigote forms of *T. cruzi*, obtained from the culture of LLC-MK_2_ cells were incubated with phosphate-buffered saline (PBS) plus 1 nM CFSE for 5 min in the dark for staining. DCs at a concentration of 1.5 × 10^5^ cells per well (24-well plate) were stimulated with saliva for 1 h. Then, the parasites were added for 18 h at a parasite-cell ratio of 3:1. After this time, the cells were collected and washed. The acquisition was made in a FACSCalibur flow cytometer (BD Immunocytometry Systems) and the analyze through the CellQuest 5.1 software (BD Biosciences) and FlowJo 10 (Tree Star Inc., Ashland, OR, USA). The supernatant of the culture was collected and the cytokines were also measured. A summary of all procedures performed and a timeline can be found in supplementary material (see Additional file 1: Table S1).

### Statistical analysis

The results were analyzed with the aid of the program GraphPad Prism 5.0 (GraphPad Software, San Diego, CA, USA). For data with a Gaussian distribution, ANOVA and a Tukey *post-hoc* test were performed; for data with a non-Gaussian distribution, a Kruskal-Wallis test with a Dunn’s *post-hoc* test were performed. Bar graphs were used to show the mean and standard error of the mean of each numerical result. The results were considered significant when the *P*-value was 0.05 (5%) or lower.

## Results

### Triatomine saliva inhibits dendritic cell differentiation

**Fig. 1:**
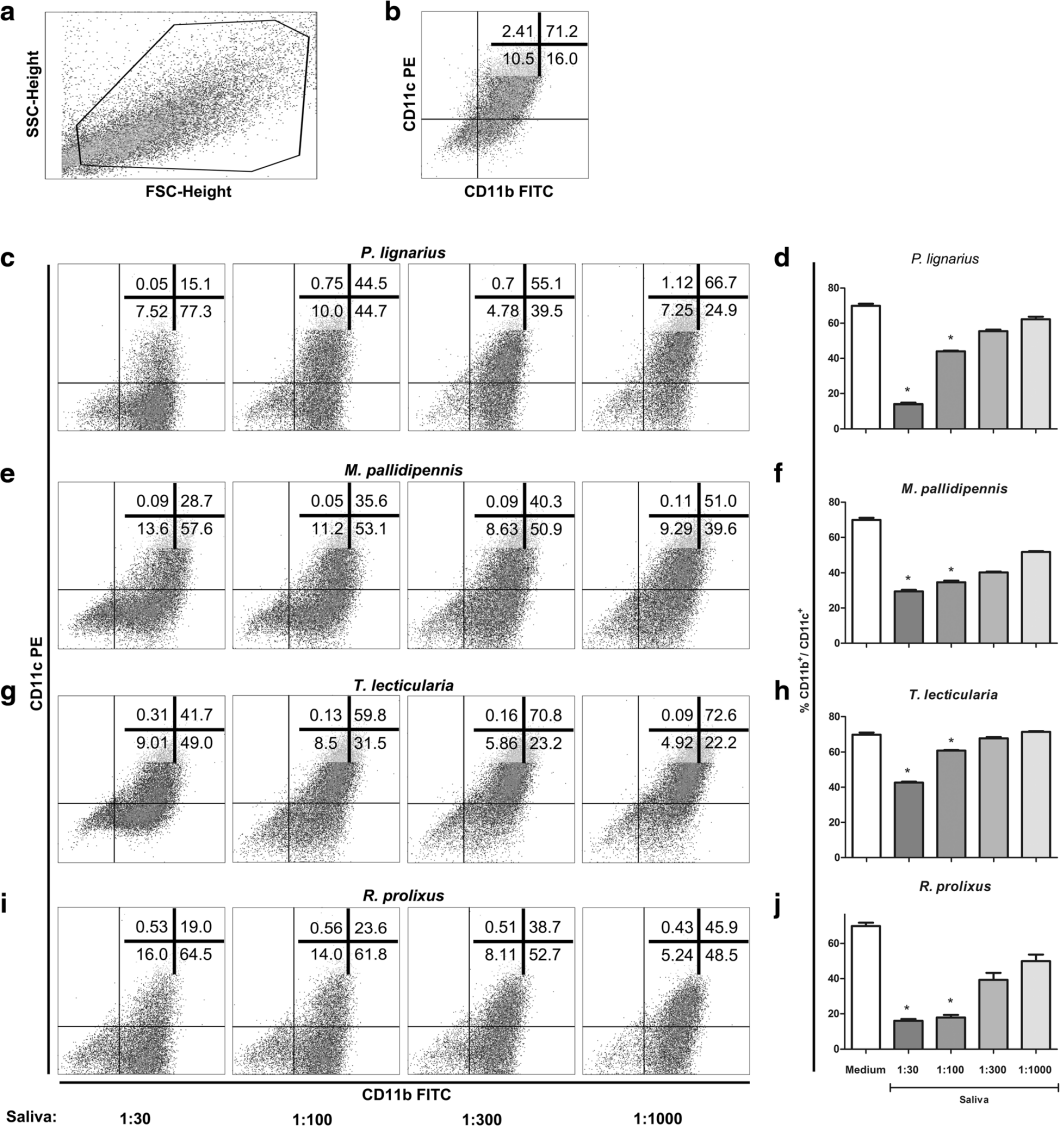
The triatomine saliva inhibits differentiation of DCs. Cells derived from bone marrow of C57BL/6 mice were cultured with GM-CSF (25 ng/ml) for 7 days in the presence or absence of triatomine saliva (*P. lignarius*, *M. pallidipennis*, *T. lecticularia* and *R. prolixus* - dilution 1:30, 1:100, 1:300 and 1:1000 v/v in the middle). On day 7, the cultured cells were collected and selected, first according to the size (FSC) and granularity (SSC) (**a**) and in sequence were evaluated for the expression of surface molecules CD11b and CD11c (**b**–**j**). **b** Cells cultured only with culture medium. Bars represent the mean ± standard error percentage of DCs expressing molecular markers from triplicate experiments. *P < 0.05 compared with cells cultured without saliva (labelled “Medium” in the graph)

The effects of triatomine saliva on the differentiation of DCs from BM precursors were studied by culturing bone marrow-derived cells in the presence of different dilutions of triatomine saliva (1:30, 1:100, 1:300 and 1:1000 v/v). Saliva of all triatomines tested significantly inhibited the differentiation of DCs (Fig. 1). When the percentage of differentiated DCs in the absence of saliva (percentage of CD11b^+^/CD11c^+^ ranging from 67.5 to 78.8) was compared with the lowest dilutions (1:30) of the saliva, it was demonstrated that there was an average reduction of CD11b^+^/CD11c^+^DCs of approximately 79% for *P. lignarius*, 57% for *M. pallidipennis*, 38% for *T. lecticularia* and 76% for *R. prolixus*. The results are representative of two independent experiments (Fig. 1). As saliva dilution had increased progressively, the effect of inhibiting the differentiation had been reduced, showing that the effect of triatomine saliva is dose-dependent. It is noteworthy that the expression of MHC-II, CD40 and CD86 molecules was also inhibited by saliva during differentiation of the DCs. The MHC-II molecule was inhibited by saliva of *P. lignarius*, *R. prolixus* and *T. lecticularia*, CD40 was inhibited by saliva of *P. lignarius*, *M. pallidipennis* and *T. lecticularia*, and the CD86 was inhibited by the saliva of *R. prolixus* (see Additional file 2: Figure S1).

### Effect of triatomine saliva on DCs that have already differentiated into CD11c^+^/CD11b^+^ cells

**Fig. 2:**
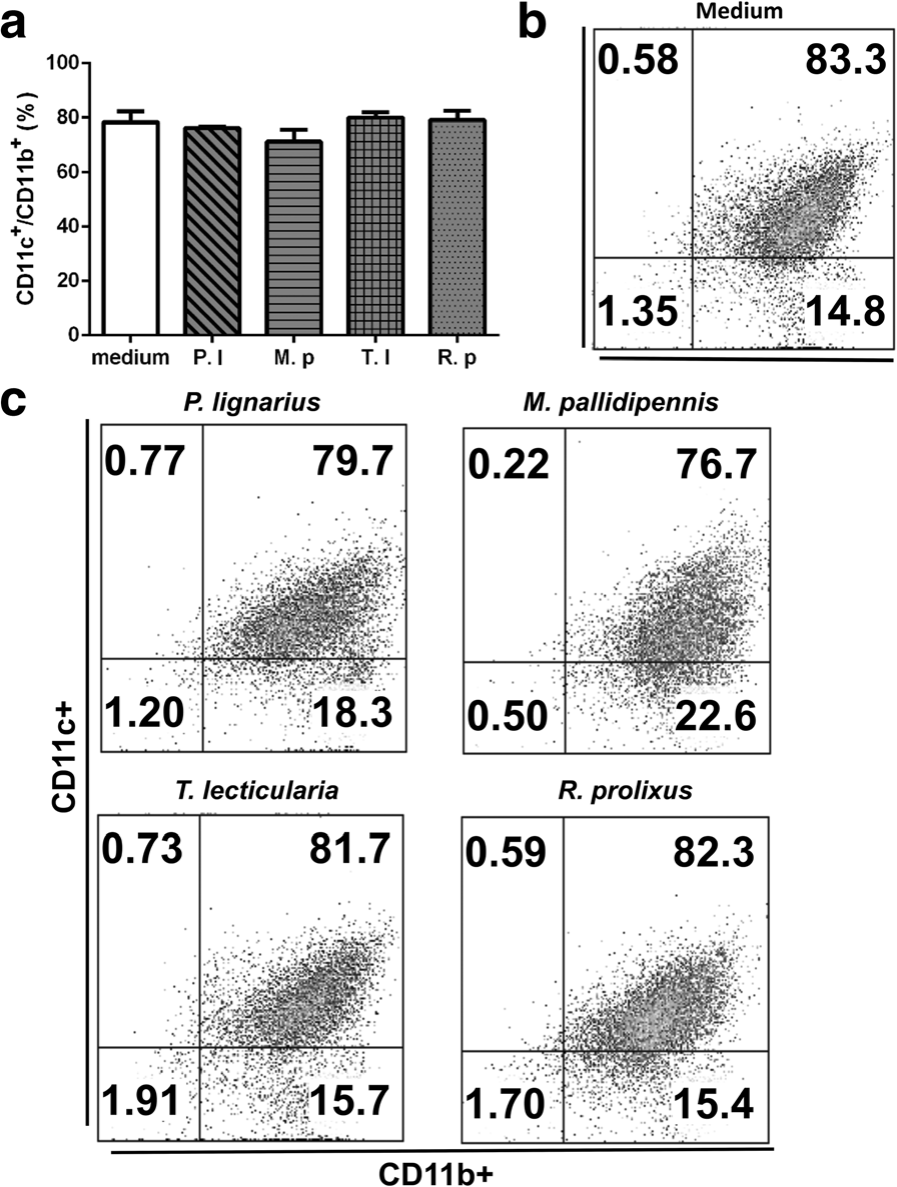
Effect of triatomine saliva on differentiated DCs. Bone marrow cells derived from C57BL/6 mice were cultured with GM-CSF (25 ng/ml) for 7 days. Immature DCs were then obtained and incubated with saliva for 18 h. The expression of CD11c and CD11b were evaluated (**a**-**c**). **b** Cells cultured only with culture medium. Bars represent the mean ± standard error percentage of DCs expressing molecular markers from duplicate experiments. The figure represents the highest concentration used. *Abbreviations*: P.l, *P. lignarius*; M.p, *M. pallidipennis*; T.l, *T. lecticularia*; R.p, *R. prolixus*

After observing the inhibitory effect of triatomine saliva on DC differentiation through the lens of cellular plasticity, where the cells have the ability to change their phenotype after differentiation, this experiment was conducted to determine whether or not saliva modulates the expression of CD11c and CD11b molecules in already differentiated DCs. The saliva of the four triatomines did not alter the expression of CD11c and CD11b molecules in these cells (Fig. 2). The DCs incubated in the absence of saliva showed an average percentage of CD11b^+^/CD11c^+^of approximately 78.35% and the DCs incubated with saliva of *P. lignarius*, *M. pallidipennis*, *T. lecticularia* and *R. prolixus* showed an average percentage of CD11b^+^/CD11c^+^ expression of 76.3, 71.2, 80.0 and 79.1%, respectively (Fig. 2). Figure 2 only demonstrates the results for DCs cultured in the presence of saliva diluted 1:30. Importantly, there was no inhibition of differentiation, independent of saliva dilution (1:30, 1:100, 1:300 and 1:1000 v/v).

### Effect of triatomine saliva on surface molecules of differentiated DCs stimulated with LPS

The DCs after activation undergo changes in expression of stimulatory and co-stimulatory molecules including MHC-II, CD40, CD80 and CD86. In order to observe whether triatomine saliva is able to interfere with this phenotype, DCs were stimulated with saliva plus LPS (100 ng/ml) and the expression of these molecules were evaluated. The expression of CD40 was inhibited in all species studied but the saliva of *M. pallidipennis* and *T. lecticularia* was able to act even in higher dilutions (1:1000) (Table 1). Furthermore, the saliva of these two species was able per se, to inhibit CD40 expression in DCs cultured in the absence of LPS stimulation (data not shown). *Panstrongylus lignarius* saliva inhibited the expression of CD40 until the dilution of 1:300 and *R. prolixus* until 1:100 (Table 1). The molecule CD80 had its expression inhibited significantly (Kruskal-Wallis H-test: *χ* ^2^ = 14.80, *df* = 4, *P* = 0.0062; *χ* ^2^ = 14.82, *df* = 4, *P* = 0.0111; and *χ* ^2^ = 4, *df* = 4, *P* = 0.0003, respectively) in the presence of saliva of *P. lignarius*, *M. pallidipennis* and *T. lecticularia* (Table 1). The *P. lignarius* saliva also inhibited significantly the molecule expression of CD86 (about 38% of inhibition) at lower dilution (1:30) (Kruskal-Wallis H-test: *χ* ^2^ = 12.10, *df* = 4, *P* = 0.0166) (Table 1), and the expression of MHC-II molecule (about 33% of inhibition) at lower dilution (1:30) (Table 1).

**Table 1:** Effect of triatomine saliva on the expression of CD40, CD80, CD86 and MHC-II on DCs stimulated with LPS

	Triatomine specie	Medium	LPS	Saliva + LPS (1:30)	Saliva + LPS (1:100)	Saliva + LPS (1:300)	Saliva + LPS (1:1000)
		Mean (SEM)	Mean (SEM)	Mean (SEM)	Mean (SEM)	Mean (SEM)	Mean (SEM)
%CD11c^+^/CD40^+^	*P. lignarius*	14.88 (1.207)	22.3 (0.3873)	16.78 (0.2926)*	16.53 (0.8189)*	15.73 (0.9207)*	21.18 (0.4871)
	*M. pallidipennis*	23.27 (0.4096)	40.7 (0.3256)	18.8 (0.07303)*	21.17 (0.3106)*	22.93 (0.2512)*	30.07 (0.9138)*
	*T. lecticularia*	23.27 (0.4096)	40.7 (0.3256)	16.45 (0.3566)*	20.47 (0.4112)*	27.45 (0.119)*	29.1 (0.4139)*
	*R. prolixus*	14.88 (1.07)	22.3 (0.3873)	19.23 (0.8557)*	19.35 (0.5923)*	20.88 (0.606)	19.93 (0.1856)
%CD11c^+^/CD80^+^	*P. lignarius*	12.37 (1.171)	17.2 (0.3801)	13.6 (0.2799)*	14.57 (0.6126)*	13.66 (0.4381)*	15.62 (0.3899)
	*M. pallidipennis*	17.75 (0.4486)	19.15 (0.2608)		15.28 (0.0821)*	16.34 (0.4987)	18.63 (0.4225)
	*T. lecticularia*	17.75 (0.4486)	19.15 (0.2608)		16.17 (0.1834)*	16.8 (0.1835)	17.18 (0.1517)
	*R. prolixus*	5.053 (0.1725)	6.33 (0.2074)	5.653 (0.6)	6.227 (0.4548)	5.89 (0.5493)	5.7 (0.7892)
%CD11c^+^/CD86^+^	*P. lignarius*	11.12 (1.306)	14.51 (1.129)	8.923 (0.27)*	10.82 (0.5174)	12.93 (0.4397)	14.55 (0.6326)
	*M. pallidipennis*	11.12 (1.306)	14.51 (1.129)	17.85 (1.893)	17.48 (0.8487)	16.74 (2.532)	14.92 (2.705)
	*T. lecticularia*	11.12 (1.306)	14.51 (1.129)	12.69 (1.371)	16.75 (1.045)	14.93 (0.573)	17.11 (0.5334)
	*R. prolixus*	8.77 (0.759)	10.35 (0.957)	12.6 (0.2887)	10.71 (1.491)	9.443 (1.071)	9.83 (0.7206)
%CD11c^+^/MHCII^+^	*P. lignarius*	14.92 (1.179)	17.09 (1.217)	11.32 (0.2409)*	14.89 (0.4705)*	18.47 (0.2345)	19.04 (0.2121)
	*M. pallidipennis*	16.74 (1.289)	18 (0.8667)	18.46 (1.591)	18.97 (0.9694)	18.39 (1.86)	18.26 (2.36)
	*T. lecticularia*	17.67 (1.408)	19.12 (1.324)	16.76 (1.58)	19.03 (0.9671)	18.4 (0.6897)	19.17 (1.056)
	*R. prolixus*	14.92 (1.179)	17.09 (1.217)	18.25 (0.4392)	17.18 (0.6218)	17.71 (0.3153)	19.09 (0.5829)

*P < 0.05 compared to cells cultured with LPS alone

### Effect of triatomine saliva on cytokine production of LPS-activated DCs

**Fig. 3:**
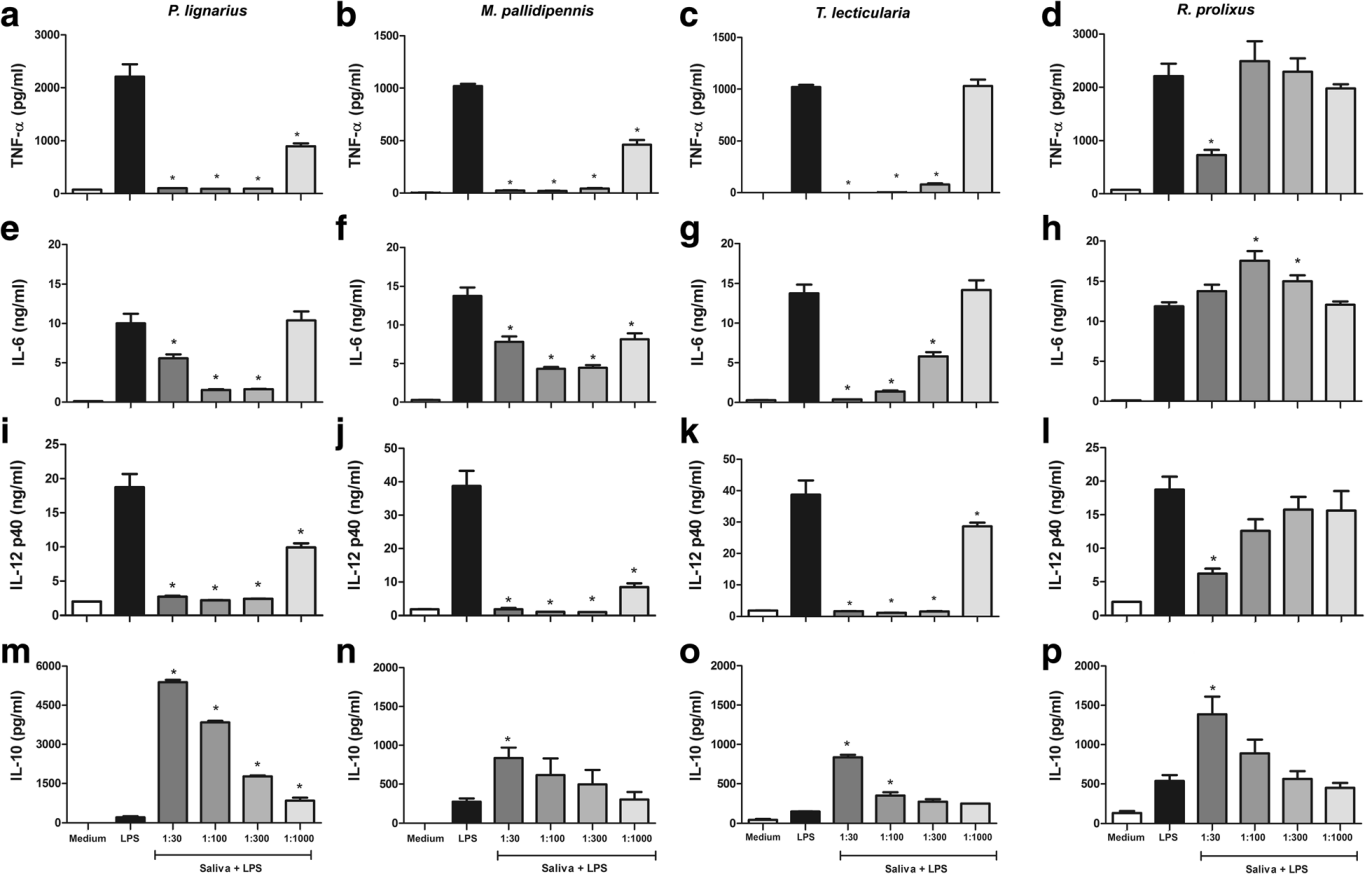
Effect of triatomine saliva in pro-inflammatory cytokines and IL-10 on DCs stimulated with LPS. Bone marrow cells derived from C57BL/6 mice were collected and cultured for 7 days in the presence of GM-CSF to allow differentiation into DCs. DCs were then pre-incubated with triatomine saliva (*P. lignarius*, *M. pallidipennis*, *T. lecticularia* and *R. prolixus*) for 1 h. DCs were then stimulated for an additional 18 h with LPS (100 ng/ml). The culture supernatant was then collected and analyzed to detect TNF-α (**a**-**d**), IL-6 (**e**-**h**), IL-12 p40 (**i**-**l**) and IL-10 (**m**-**p**) by ELISA. Bars represent the mean ± standard error production level of TNF-α, IL-12 p40, IL-6 and IL-10 in cultured DCs from triplicate experiments. *P < 0.05 compared with DCs cultured with LPS

Saliva of all triatomines inhibited significantly (ANOVA: *F*_(4,14)_ = 67.12, *P* < 0.0001; *F*_(4,23)_ = 305.4, *P* < 0.0001; *F*_(5,33)_ = 31.47, *P* < 0.0001; *F*_(4,15)_ = 8.87, *P* = 0.0007) TNF-α production in LPS stimulated DCs (Fig. 3a-d). *Panstrongylus lignarius* and *M. pallidipennis* saliva inhibited the production of TNF-α at all dilutions tested (ANOVA: *F*_(4,14)_ = 67.12, *P* < 0.0001; *F*_(4,23)_ = 305.4, *P* < 0.0001) (Fig. 3a, b, respectively). In the dilutions of 1:30, 1:100 and 1:300 the inhibition was greater than 95% reaching the levels produced by cells without stimulation and at the dilution of 1:1000 the inhibition was greater than 50% (ANOVA: *F*_(4,14)_ = 67.12, *P* < 0.0001; *F*_(4,23)_ = 305.4, *P* < 0.0001) (Fig. 3a, b). The saliva from *T. lecticularia* inhibited the production of TNF-α until the dilution 1:300, with inhibition values greater than 80% and the saliva from *R. prolixus* was capable of inhibiting about 67% of this production but only at lower dilution (1:30) (Fig. 3 c, d, respectively). The cytokine IL-6 was inhibited significantly (ANOVA: *F*_(4,15)_ = 30.64, *P* < 0.0001; *F*_(4,15)_ = 29.92, *P* < 0.0001; *F*_(4.14)_ = 82.36, *P* < 0.0001) by saliva of *P. lignarius*, *M. pallidipennis* and *T. lecticularia*, respectively (Fig. 3 e-g). An interesting fact was that the saliva of *P. lignarius* and *M. pallidipennis* were similar in having the highest level of inhibition at a dilution of 1:100 and not with the lower dilution, 1:30 (Fig. 3e, f, respectively). *Rhodnius prolixus* saliva showed the opposite effect when compared with other saliva stimulating the production of IL-6 cytokine (dilutions 1:100 and 1:300) (Fig. 3h). It is noteworthy that these results are representative of at least two independent experiments.

The production of the cytokine IL-12p40 was significantly inhibited (ANOVA: *F*_(4,15)_ = 62.54, *P* < 0.0001; *F*_(4,15)_ = 59.83, *P* < 0.0001; *F*_(4,15)_ = 77.16, *P* < 0.0001; *F*_(4,14)_ = 6.883, *P* = 0.0028) for all evaluated saliva (Fig. 3 i-l). Saliva of *P. lignarius*, *M. pallidipennis* and *T. lecticularia* exerted a strong inhibitory role in inhibiting IL-12p40 production in all dilutions used, so that averages of inhibition were higher than 80% in the three smaller dilutions tested (1:30, 1:100 and 1:300) (Fig. 3 i-k). The saliva of *R. prolixus* was able to significantly inhibit (ANOVA: *F*_(4,14)_ = 6.883, *P* = 0.0028) the production of IL-12p40 only at lower dilution (1:30) with the mean percentage inhibition of approximately 66% (Fig. 3l). The saliva of all species studied was able to increase significantly (ANOVA: *F*_(4,25)_ = 936.1, *P* < 0.0001; *F*_(4,28)_ = 2.880, *P* = 0.0408; Kruskal-Wallis H-test: *χ* ^2^ = 20.57, *df* = 4, *P* = 0.0004; ANOVA: *F*_(4,33)_ = 7.930, *P* = 0.0001) IL-10 production (Fig. 3m-p). The saliva of *M. pallidipennis* and *R. prolixus* increased significantly (ANOVA: *F*_(4,28)_ = 2.880, *P* =0.0408; and *F*_(4,33)_ = 7.930, *P* = 0.0001, respectively) in the lowest dilution tested, with an increase of 3-fold and 4-fold, respectively (Fig. 3n, p, respectively). Saliva of *T. lecticularia* was able to stimulate the production of IL-10 until the dilution of 1:100, and in the lower dilution, the increase was about 5-fold compared to DCs stimulated just with LPS (Fig. 3o). Surprisingly, the saliva of *P. lignarius* stimulated IL-10 production at all concentrations and in the low dilution. The increase was about 25-fold compared to DCs stimulated just with LPS (Fig. 3m).

### Saliva *per se* modulates the production of cytokines in DCs

**Fig. 4:**
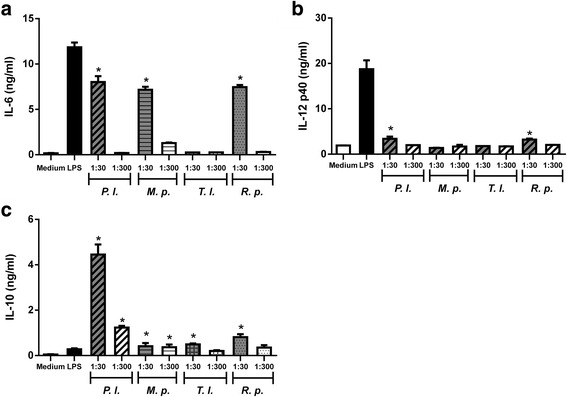
Effect of triatomine saliva in cytokine production of DCs without LPS. Bone marrow cells derived from C57BL/6 mice were collected and cultured for 7 days in the presence of GM-CSF to allow differentiation into DCs. DCs were then pre-incubated with triatomine saliva (*P. lignarius*, *M. pallidipennis*, *T. lecticularia* and *R. prolixus*) for 18 h. The culture supernatant was then collected and analyzed to detect IL-6 (**a**), IL-12p40 (**b**), IL-10 (**c**) and TNF-α (data not shown) by ELISA. Bars represent the mean ± standard error production level of TNF-α, IL-12 p40, IL-6 and IL-10 in cultured DCs from triplicate experiments. *P < 0.05 compared with DCs cultured with medium. *Abbreviations*: P.l, *P. lignarius*; M.p, *M. pallidipennis*; T.l, *T. lecticularia*; R.p, *R. prolixus*

In addition to evaluating the effect of triatomine saliva on DCs stimulated with LPS, it was sought to assess whether the saliva would be able, per se, to modulate the production of cytokines in immature DCs without LPS stimulation. The cytokine IL-6 production was increased significantly (Kruskal-Wallis H-test: *χ* ^2^ = 48.07, *df* = 8, *P* < 0.0001) in the lower dilutions (1:30) for three of the four tested saliva (except for *T. lecticularia*) (Fig. 4a). Regarding the cytokine IL-12p40, a significant (Kruskal-Wallis H-test: *χ* ^2^ = 31.03, *df* = 8, *P* = 0.0001) induction in their production was observed when the cells were cultured with saliva (diluted 1:30) of *P. lignarius* and *R. prolixus* (Fig. 4b). It is very important to highlight that the production of IL-12p40 stimulated by the saliva was present only when the cells were stimulated with saliva in the lower dilution (1:30) and this induction was much lower compared with that in the cells stimulated by LPS (Fig. 4b). The saliva of all species, *per se*, was capable of significantly stimulating (Kruskal-Wallis H-test: *χ* ^2^ = 42.27, *df* = 8, *P* < 0.0001) IL-10 production and this effect was significant even when the cells were incubated with saliva diluted 1:300, except *T. lecticularia* and *R. prolixus,* which have significant inhibition just with the lowest dilution used (1:30) (Kruskal-Wallis H-test: *χ* ^2^ = 42.27, *df* = 8, *P* < 0.0001) (Fig. 4c). In relation to the cytokine TNF-α, neither of the species caused significant changes in its production (data not shown).

### Effect of triatomine saliva on DC viability in vitro

**Fig. 5:**
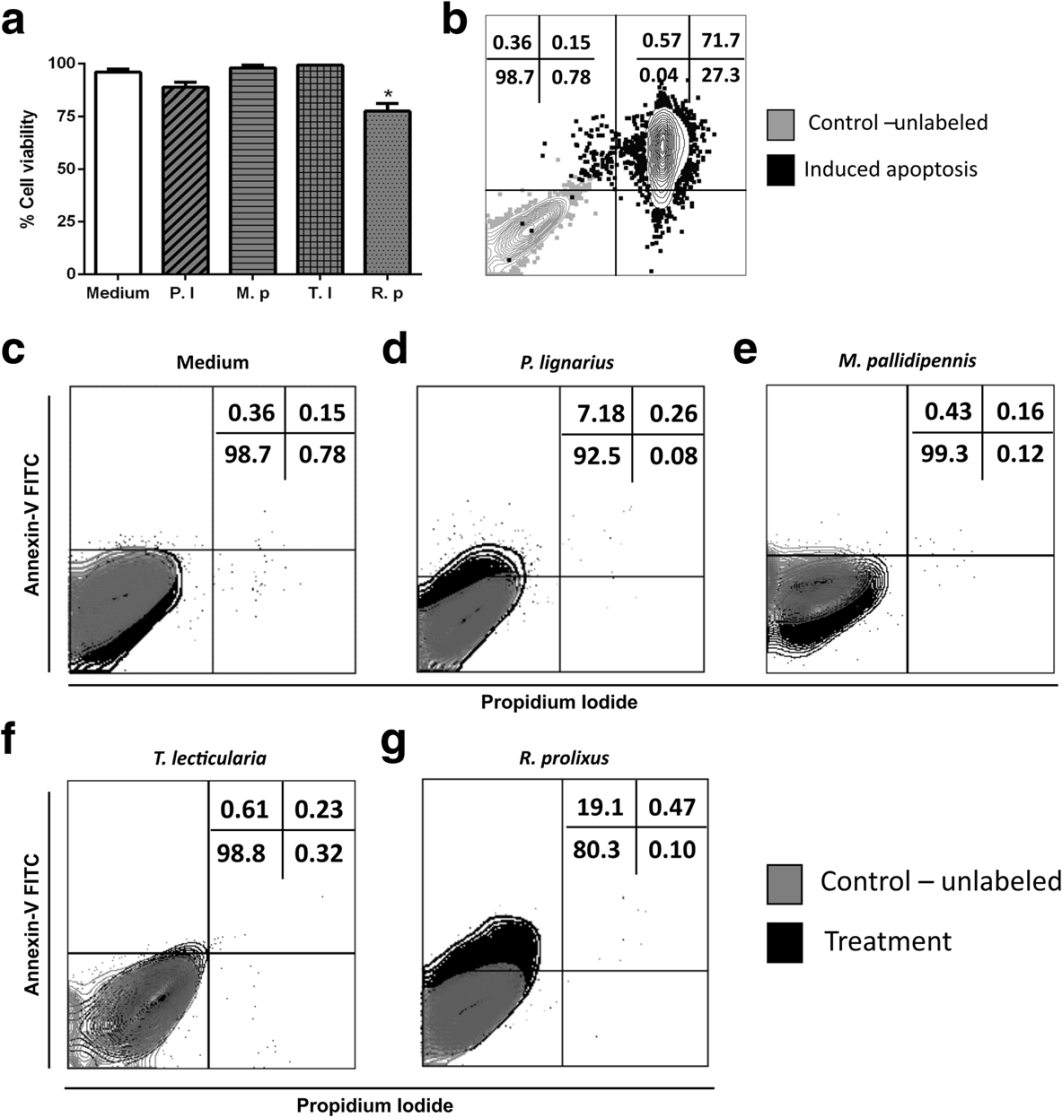
Effect of triatomine saliva on DC viability in vitro. Bone marrow cells derived from C57BL/6 mice were collected and cultured for 7 days in the presence of GM-CSF to allow differentiation into DCs. DCs were then incubated with triatomine saliva (*P. lignarius*, *M. pallidipennis*, *T. lecticularia* and *R. prolixus* - dilution 1:30 v/v in the middle) for 18 h. The cells were then collected and stained with Annexin V and propidium iodide to evaluate apoptosis by flow cytometry. **a** Graphical representation of the cell viability, the bars represent mean ± standard error (SEM) of triplicates. **b** Comparison between the positive control of cell viability and apoptosis induced by heat. **c** DCs incubated just with medium, normal cell viability. **d**-**g** DCs incubated with triatomine saliva (*P. lignarius*, *M. pallidipennis*, *T. lecticularia* and *R. prolixus* - dilution 1:30 v/v in the middle, respectively).*P < 0.05 compared with CD cultured with medium. The figures are representative of two independent experiments. Bars represent the mean ± SEM number of viable DCs from duplicate experiments. *Abbreviations*: P.l, *P. lignarius*; M.p, *M. pallidipennis*; T.l, *T. lecticularia*; R.p, *R. prolixus*

To assess whether the saliva of different triatomines alter DC viability, an apoptosis test was performed. Saliva of *P. lignarius, M. pallidipennis* and *T. lecticularia* were not able to induce apoptosis of DCs even when the lower dilution of saliva was used (1:30 v/v) (Fig. 5a). The saliva of *R. prolixus* was the only one that induced apoptosis (Annexin V^+^) of DCs significantly (Kruskal-Wallis H-test: *χ* ^2^ = 17.81, *df* = 4, *P* = 0.0013), reaching a percentage mean inhibition of 16% when compared to the positive control (Annexin V^-^ PI^-^) (Fig. 5a, g). It is important to highlight that although Fig. 5 shows only the results with saliva diluted 1:30, the saliva from the four species was tested in all dilutions described in this study.

### Effect of triatomine saliva on *T. cruzi* infection in DCs

**Fig. 6:**
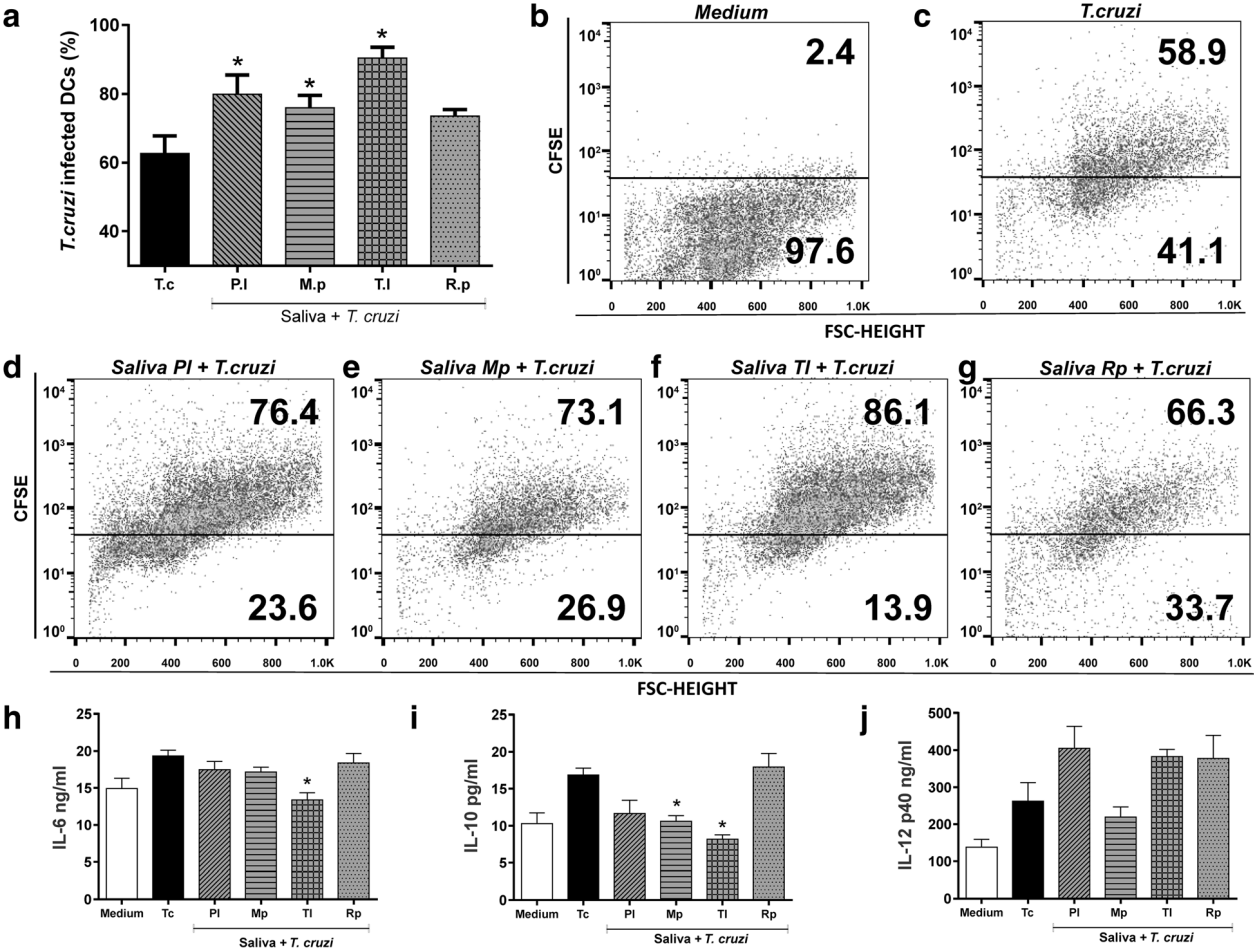
Effect of triatomine saliva on cytokine production and *T. cruzi* infection in DCs. Murine bone marrow-derived DCs were incubated with saliva (1:100 v/v) and CFSE-labeled *T. cruzi* (Y strain) (1 nM CFSE; at a parasite-cell ratio of 3:1) and fluorescent DCs were quantified by flow cytometry after 18 h of culture (**a**-**g**). **a** Percent of *T. cruzi*-infected DCs. **h**-**j** The culture supernatant was collected and analyzed to detect IL-6 (**h**), IL-12 p40 (**i**), and IL-10 (**j**) by ELISA. Bars represent the mean ± standard error of the invasion and cytokine production in cultured DCs. *P < 0.05 compared with DCs cultured with *T. cruzi* only. *Abbreviations*: P.l, *P. lignarius*; M.p, *M. pallidipennis*; T.l, *T. lecticularia*; R.p, *R. prolixus*

To evaluate the effect of triatomine saliva on *T. cruzi* infection, DCs were incubated with different triatomine saliva and infected by *T. cruzi* CFSE^+^. The experiments demonstrated that the “Y” strain of *T. cruzi* in the absence of saliva infects nearly 60% of the DCs and in the presence of triatomine saliva the invasion by *T. cruzi* was higher (Fig. 6a-f). More specifically, the DCs cultured with saliva of *P. lignarius*, *M. pallidipennis* and *T. lecticularia* had a significant increase (ANOVA: *F*_(4,20)_ = 26.17, *P* < 0.0001) of the invasion by *T. cruzi*, between 14 and 30%. The only saliva that did not induce increased levels of DC invasion by *T. cruzi* was that of *R. prolixus* (Fig. 6a, g). Regarding cytokines, the results show that DCs infected with *T. cruzi* plus saliva of *T. lecticularia* decrease the production of the cytokine IL-6 and the saliva of *M. pallidipennis* and *T. lecticularia* decrease the production of the cytokine IL-12p40, when compared with cells that were cultured with *T. cruzi* only (Fig. 6h, i, respectively). Still, despite saliva of three species inducing increased IL-10 production in DCs cultured with *T. cruzi*, this increase was not statistically significant (Fig. 6j).

### Triatomine saliva does not contain prostaglandin E_2_ (PGE_2_)

Seeking to determine the possible molecule responsible for the observed effects and based on data in the literature, which demonstrated a relationship between effects similar to those described and the presence of PGE_2_ in the saliva of other arthropods [18, 20, 21], the presence of PGE_2_ in the saliva of the triatomine species was evaluated and was not detected through the methodology used.

## Discussion

The present study demonstrated that triatomine saliva modulates the differentiation and maturation of DCs and these effects may be independent of PGE_2_, moreover that triatomine saliva increases *T. cruzi* invasion in this cells (see Additional file 3: Table S2). These results are very important because they demonstrate for the first time that triatomines are able to modulate the DC biology and *T. cruzi* invasion, with the aggravating factor that the effects observed here were found even in high dilutions of saliva, except for *R. prolixus.* These findings add knowledge of the potential effect of triatomine saliva, since so far, with rare exceptions, only studies on anti-hemostatic activity of triatomine saliva had been reported [3, 4].

The four species of triatomine used belong to the four main genera involved in the transmission of *T. cruzi* and originale from different habitats [22–24]. In this context, evolutionary and gender differences may help explain the variation in the results of this work. During evolution, arthropod vectors feed on different hosts and because of this, they are challenged by various defense systems and subjected to different evolutionary pressures which may induce them to produce and secrete distinct bioactive salivary molecules with singular modulatory properties or in distinct concentrations. It is noteworthy that *R. prolixus* has only one pair of salivary glands while the other three species studied here have three pairs of glands and these have different molecular compositions depending on the assessed pair [25]. It is worth mentioning also that the biological effects of saliva demonstrated in this work do not show correlation with the protein concentration of saliva and therefore, after pilot experiments, only dilutions fixed for all species were evaluated, instead of doing the analyses defined by protein concentration.

Regarding DC biology, it is shown for the first time that saliva from different triatomine species was able to inhibit the DC differentiation. Monocytes recruited to the skin may differ locally on DCs [26]. Thus, this local inhibition of differentiation by saliva would be of great importance as it would hamper the DC repopulation. In agreement with our findings, tick saliva from *Rhipicephalus sanguineus* and *Amblyomma cajennense* also were able to inhibit DC differentiation [21, 27]. In all cases, the effects found were due to whole saliva or PGE_2_. Recently a novel protein was described, called Japanin, derived from the saliva of the tick *Rhipicephalus appendiculatus*, with inhibitory activity on DC differentiation [28]. Although it seems a standard effect for blood-sucking arthropods, saliva’s ability to inhibit the differentiation of DCs is not common to all of them, e.g. *Aedes aegypti* mosquito saliva is not able to inhibit the differentiation of these cells [29].

In addition to the effects on DC differentiation, our findings show that saliva also altered the expression of MHC-II, CD40 and CD86 in these cells differentiated in the presence of saliva. This means that even the cells that could differentiate into DCs will not be able to trigger an effective antigen presentation. Slightly different results with the saliva of the *R. sanguineus* tick were observed, as MHC-II and CD40 did not have their expression modified, and only CD86 had the expression inhibited [27].

Once the inhibitory effect of saliva on DC differentiation was verified, it was investigated whether the saliva also had the ability to change the differentiation markers (CD11c and CD11b) of the differentiated-DCs. None of the four species was able to change the CD11c^+^/CD11b^+^ phenotype of differentiated DCs. Unlike what was observed in our studies, it is known that saliva of the tick *R. sanguineus* decreases the expression of CD11b and CD11c on DCs already differentiated generating a cell population CD11c^−^/CD11b^−^ or CD11c^−^/CD11b^+^ [27]. Thus, it can be deduced that, unlike suggested for *R. sanguineus* ticks [27, 30], during triatomine saliva inoculation DCs of the skin retain their phenotype.

In an attempt to clarify the impact of saliva on DC maturation, cytokine production and molecule expression were evaluated in LPS-activated DCs. The saliva of the four species evaluated was able to inhibit the production of pro-inflammatory cytokines. The observed events are quite relevant because IL-12 and TNF-α are cytokines related with the induction of inflammation and mounting of a Th1-type response profile, hostile environment for vectors and pathogens transmitted by them [31]. *Rhodnius prolixus* did not show strong inhibition in cytokine production but it is already known that this species has its saliva constituted of nitrophorins as the most abundant molecules [32] and so far no activity involving DCs was attributed to these molecules. It is noteworthy that the saliva of *R. prolixus* induced 16% of apoptosis and this could explain some of the *R. prolixus* inhibition only in dilution 1:30.

Regarding IL-10 cytokine, saliva of all four species increased their production, but each one with a different intensity. *Panstrongylus lignarius* increased IL-10 production approximately 25-fold more than the other species, suggesting that this saliva has more molecules or has more potent molecules to stimulate such production. IL-10 is well known for its immunosuppressive effect [33] and like other blood-sucking arthropods, triatomines stimulate its production. Also, IL-10 production is quite relevant since several of the events observed may be a consequence of this production. Previous reports describe this cytokine inhibiting the production of pro-inflammatory cytokines, decreasing the MHC-II expression, decreasing expression of CD80, inhibiting the antigenic presentation, inhibiting the production of IL-12, and by blocking the differentiation of DCs [34]. In summary, triatomine saliva inhibiting pro-inflammatory cytokines also induces anti-inflammatory response.

The cytokine production was also evaluated in immature DCs, i.e. without LPS stimulation. In these cells, the triatomine saliva stimulated IL-12 and IL-6 production, results quite different from LPS-activated DCs, where saliva inhibited the cytokine production. A similar effect was observed when LPC was used [35]. This molecule in presence of LPS counteracts some of the TLR-mediated intracellular responses, like the production of pro-inflammatory cytokines, overcoming in an anti-inflammatory environment. However, when added separately, both LPS and LPC might be signaling through TLR-4, triggering a pro-inflammatory phenotype. Until so far, LPC was just described in *R. prolixus* saliva [12, 13]. In our results, it is important to highlight that the induction of IL-12 and IL-6 production by saliva was much lower than by LPS, a classical TLR ligand. Regarding IL-10, the saliva effect in immature DCs was the same observed in LPS-activated DCs.

The mechanisms which these triatomines use to modulate cytokine production are not well known and the possibilities for inhibiting production of certain cytokines could be blocking the interaction between LPS and TLR-4. Another possibility would be the interaction of any molecule of saliva with some intracellular factors, as noted, for example, in the protozoan *Toxoplasma gondii* that modulates intracellular signaling pathways to facilitate its survival in the host [36]. Accordingly, a good example that explains the inhibition of signaling pathways are LPC (already described for saliva of *R. prolixus*) and saliva of *R. sanguineus* ticks that inhibit MAP-kinases ERK-1/2 and/or p38 which are essential for the activation of various cell populations [37].

The activation of T lymphocytes is dependent on a set of interactions involving the expression of MHC-II, CD40, CD86 and CD80 molecules in DCs, and in the absence of any of the signals necessary for T cell activation this process is hindered, and the T lymphocyte enter in a state of cell anergy [38]. Our results showed different effects in stimulatory and co-stimulatory molecules. In view of that, modulation in the expression of these molecules by triatomine saliva along with reducing the production of pro-inflammatory and induction of anti-inflammatory cytokines may have great importance for the success of parasitism of the triatomines and/or transmission of pathogens via saliva. In agreement with our results, several other studies have shown that other arthropods’ saliva has modulatory effects on the expression of stimulatory and co-stimulatory molecules and production of pro-inflammatory cytokines in DCs [18, 20, 27, 28, 39, 40].

Another way by which parasites modulate DCs is through apoptosis induction, as shown for some organisms like viruses and bacteria [41, 42]. The results with triatomine saliva showed that among the four species studied, only the saliva of *R. prolixus* was able to induce apoptosis under the conditions tested. Probably, this effect is due to the presence of LPC in the saliva of *R. prolixus* [12] since this molecule is also able to induce cell death by apoptosis [43]. The lack of induction of apoptosis by the saliva of the other species of triatomine suggests that they have no LPC in the saliva or have it in low concentrations. Given that the saliva of *R. prolixus* modulates many steps on DC biology (differentiation, maturation and cytokine production) at different intensities we believe these effects may be caused by LPC-dependent apoptosis, by other bioactive molecules, or the sum of these effects.

The modulation by tick saliva on DCs, such as IL-12 and TNF-αinhibition, and IL-10 stimulation, may be caused due to the presence of PGE_2_ in saliva [18, 20, 21]. PGE_2_ is a lipid with various immunomodulatory activities and acts on different immune cells. In DCs, among other effects, it inhibits differentiation and pro-inflammatory cytokines production and stimulates IL-10 synthesis [44–46]. In our experiments, significant concentrations of PGE-_2_ in saliva were not detected, so the observed effects showed here for triatomines may occur regardless of the presence of this molecule. Despite this, PGE_2_ effect cannot be totally ruled out, as triatomine saliva can contain molecules able to activate the PGE_2_ production in the host cells, such as with *Lutzomyia longipalpis* saliva, that stimulates PGE_2_ production in macrophages [14]. It is important to note that the absence of PGE_2_ in saliva open the possibility to investigate other molecules in saliva that are able to exert immunomodulatory effects. For example, studies evaluating the genome or sialome of triatomines demonstrated that their saliva presents a great number of immunomodulatory proteins of distinct families including ixostatins, kunitz, lipocalins and serine protease inhibitors [3, 17]. Future studies must be done to test this hypothesis.

*Trypanosoma cruzi* needs to hide into cells in order to be spread and to avoid the host immune system. The triatomine saliva enhances in vivo *T. cruzi* infection, increasing 5-fold the association of *T. cruzi* with macrophages and increasing up to 6-fold the blood parasitemia [12]. Here, our results showed that triatomine saliva was able to increase *T. cruzi* invasion also in DCs. These cells could have an important role in *T. cruzi* infection, acting like a “Trojan horse”. Regarding the cytokine production in *T. cruzi-*infected DCs, we observed different results compared with the experiments with LPS-stimulated DCs. Only saliva of *M. pallidipennis* and *T. lecticularia* have inhibitory effects on *T. cruzi*-infected DCs*.* In this sense, we need to observe that *T. cruzi* invasion and maintenance in DCs triggers other intracellular pathways than LPS signal pathway [47, 48] and the saliva does not seem to be able to modulate all these different pathways.

## Conclusions

Taken together, the present results demonstrated that saliva of triatomines possesses molecules capable of modulating the host immune system by acting on DCs from differentiation to maturation, moreover helping *T. cruzi* invasion. Thus, these results shed more light on triatomine-host interactions, the mechanisms by which triatomines downregulate host immune response, possibly favoring susceptibility to infestations, and provide new perspectives for the discovery of new molecules derived from blood-feeding arthropods with potential therapeutic uses.

## Additional files

Additional file 1: Table S1. Timeline and procedures performed. Timeline showing the approaches used to assess the in vitro effect of saliva of different triatomine species on the biology of dendritic cells. (DOCX 15 kb)
Additional file 2: Figure S1. The triatomine saliva inhibits MHC-II, CD40 and CD86 expression in DCs differentiated in the presence of saliva. Cells derived from bone marrow of C57BL/6 mice were cultured with GM-CSF (25 ng/ml) for 7 days in the presence or absence of triatomine saliva (*P. lignarius*, *M. pallidipennis*, *T. lecticularia* and *R. prolixus* - dilution 1:30, 1:100, 1:300 and 1:1000 v/v in the middle). On the day 7, the cultured cells were collected and were evaluated for the expression of surface molecules MHC-II (a-d), CD40 (e-h) and CD86 (i-l). Bars represent the mean ± standard error percentage of DCs expressing molecular markers from triplicate experiments. * *P* < 0.05 compared with cells cultured without saliva (labelled “Medium” in the graph). (TIF 2486 kb)
Additional file 3: Table S2. Summary of saliva effects on different phenotypic and functional features of dendritic cells. Summary of the effects of saliva on the maturation, differentiation, expression of surface molecules, cytokine production, viability and susceptibility to *Trypanosoma cruzi* invasion. The arrows correspond to the significant dilution of saliva used. *Key*: Up-arrow is upregulation, down-arrow is downregulation or inhibition. *Abbreviations*: n/a: not altered; n/d: not detected. (DOCX 16 kb)

## Abbreviations

CD: Cluster of differentiation
DCs: Dendritic cells
GM-CSF: Granulocyte-macrophage colony-stimulating factor
IL: Interleukin
LPC: Lysophosphatidylcholine
LPS: Lipopolysaccharid
PGE-_2_: Prostaglandin-E_2_
TLR: Toll-like receptor
