# Supplementary material for: Effect of the saliva from different triatomine species on the biology and immunity of TLR-4 ligand and Trypanosoma cruzi-stimulated dendritic cells
Source: Parasit Vectors. 2016 Dec 9;9:634. doi: 10.1186/s13071-016-1890-x (PMC5148907; doi:10.1186/s13071-016-1890-x)
Supplement: Additional file 1: Table S1. — Timeline and procedures performed. Timeline showing the approaches used to assess the in vitro effect of saliva of different triatomine species on the biology of dendritic cells. (DOCX 15 kb) [file 13071_2016_1890_MOESM1_ESM.docx]

| ***Timeline*** | | | | | |
| --- | --- | --- | --- | --- | --- |
| ***Day 0*** | ***Day 3*** | ***Day 4*** | ***Day 7*** | | ***Day 8*** |
| DC differentiation (BM cells + GM-CSF + saliva) | Addition of saliva | Addition of GM-CSF | *Determination of the effect of saliva on the differentiation of DCs (CD11c, CD11b, MHC-II, CD40 and CD86)* | | - |
|  |  |  |  |  |  |
| DC differentiation (BM cells + GM-CSF) | - | Addition of GM-CSF | Check-point for DC differentiation (CD11b and CD11c) | Addition of saliva (18 h incubation) | *Determination of the effect of saliva on the expression of CD11b and CD11c and cytokine production in differentiated DCs* |
|  |  |  |  |  |  |
|  |  |  |  |  | *Determination of apoptosis* |
|  |  |  |  | Addition of LPS or saliva + LPS (1h + 18 h incubation) | *Determination of the effect of saliva on the maturation of DCs (MHC-II, CD40, CD80 and CD86). Collection of supernatant to measure the cytokines, IL-12p40, IL-6, IL-10, and TNF-α* |
|  |  |  |  |  |  |
|  |  |  |  |  |  |
|  |  |  |  | Addition of *T. cruzi* or saliva + *T. cruzi* (18 h incubation) | *Determination of T. cruzi Invasion and cytokine production* |
|  |  |  |  |  |  |
|  |  |  |  |  |  |
