# Supplementary figures and images for: Effect of the saliva from different triatomine species on the biology and immunity of TLR-4 ligand and Trypanosoma cruzi-stimulated dendritic cells
Source: Parasit Vectors. 2016 Dec 9;9:634. doi: 10.1186/s13071-016-1890-x (PMC5148907; doi:10.1186/s13071-016-1890-x)

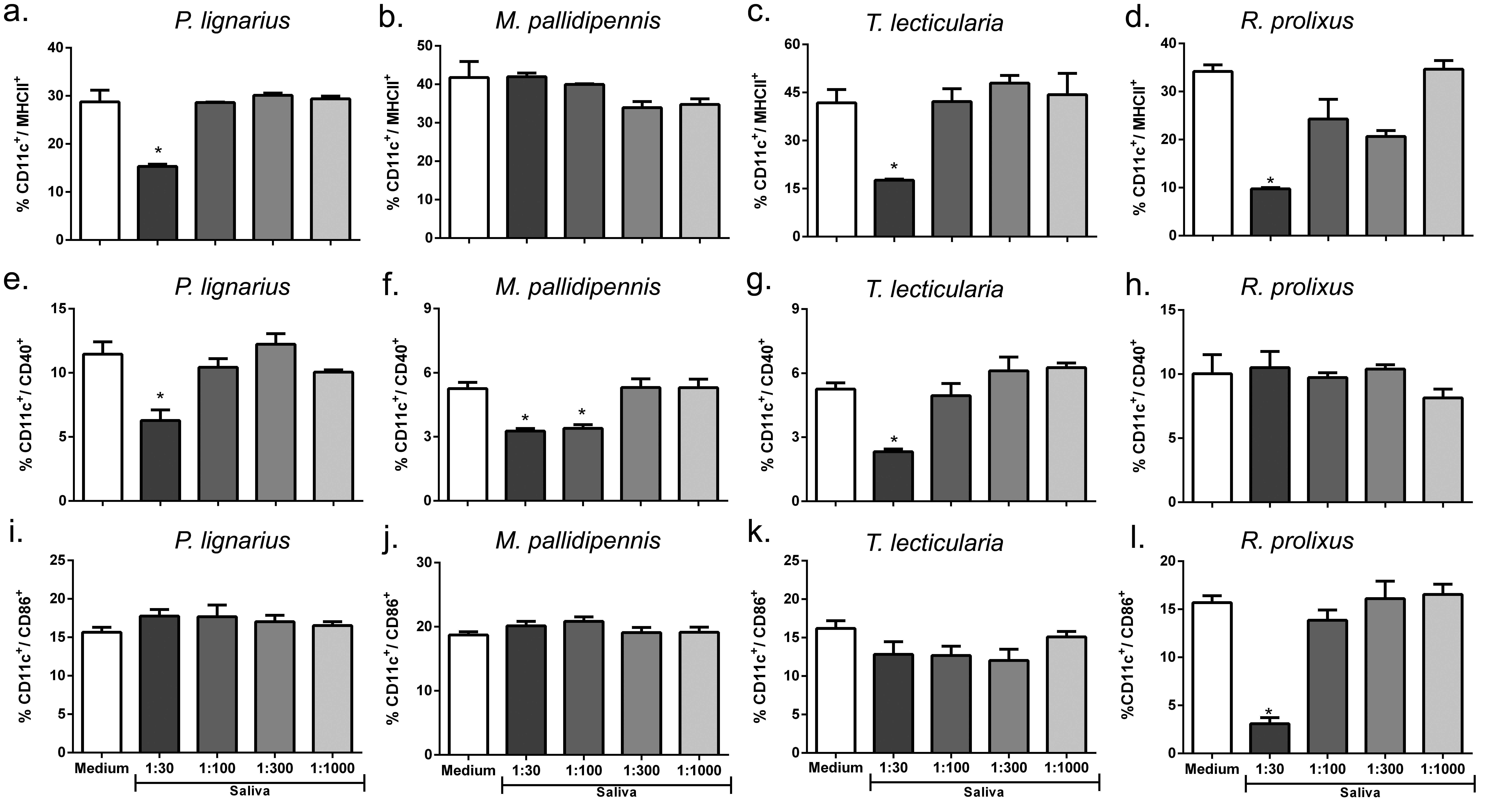

Supplement: Additional file 2: Figure S1. — The triatomine saliva inhibits MHC-II, CD40 and CD86 expression in DCs differentiated in the presence of saliva. Cells derived from bone marrow of C57BL/6 mice were cultured with GM-CSF (25 ng/ml) for 7 days in the presence or absence of triatomine saliva (P. lignarius, M. pallidipennis, T. lecticularia and R. prolixus - dilution 1:30, 1:100, 1:300 and 1:1000 v/v in the middle). On the day 7, the cultured cells were collected and were evaluated for the expression of surface molecules MHC-II (a-d), CD40 (e-h) and CD86 (i-l). Bars represent the mean ± standard error percentage of DCs expressing molecular markers from triplicate experiments. * P < 0.05 compared with cells cultured without saliva (labelled “Medium” in the graph). (TIF 2486 kb) [file 13071_2016_1890_MOESM2_ESM.tif]
