# Supplementary material for: Effect of the saliva from different triatomine species on the biology and immunity of TLR-4 ligand and Trypanosoma cruzi-stimulated dendritic cells
Source: Parasit Vectors. 2016 Dec 9;9:634. doi: 10.1186/s13071-016-1890-x (PMC5148907; doi:10.1186/s13071-016-1890-x)
Supplement: Additional file 3: Table S2. — Summary of saliva effects on different phenotypic and functional features of dendritic cells. Summary of the effects of saliva on the maturation, differentiation, expression of surface molecules, cytokine production, viability and susceptibility to Trypanosoma cruzi invasion. The arrows correspond to the significant dilution of saliva used. Key: Up-arrow is upregulation, down-arrow is downregulation or inhibition. Abbreviations: n/a: not altered; n/d: not detected. (DOCX 16 kb) [file 13071_2016_1890_MOESM3_ESM.docx]

|  | **Parameter** | ***P. herreri*** | ***M. pallidipennis*** | ***T. lecticularia*** | ***R. prolixus*** |
| --- | --- | --- | --- | --- | --- |
| **Evaluation of the effect of saliva on processes of the differentiation of DCs** | CD11b^+^/CD11c^+^ | ↓ ↓ | ↓ ↓ | ↓ ↓ | ↓ ↓ |
|  | MHC-II | ↓ | N/A | ↓ | ↓ |
|  | CD40 | ↓ | ↓ ↓ | ↓ | N/A |
|  | CD86 | N/A | N/A | N/A | ↓ |
| **Evaluation of the effect of saliva in already differentiated DCs** | CD11b^+^/CD11c^+^ | N/A | N/A | N/A | N/A |
|  | IL-6 | ↑ | ↑ | N/A | ↑ |
|  | IL-12p40 | ↑ | N/A | N/A | ↑ |
|  | IL-10 | ↑ ↑ | ↑ ↑ | ↑ | ↑ |
| **Evaluation of DCs apoptosis** | Annexin V^+^ | N/A | N/A | N/A | + |
| **Evaluation of the effect of saliva on LPS-activated DCs** | MHC-II | ↓ ↓ | N/A | N/A | N/A |
|  | CD40 | ↓ ↓ ↓ | ↓ ↓ ↓ ↓ | ↓ ↓ ↓ ↓ | ↓ ↓ |
|  | CD80 | ↓ ↓ ↓ | ↓ | ↓ | N/A |
|  | CD86 | ↓ | N/A | N/A | N/A |
|  | TNF-α | ↓ ↓ ↓ ↓ | ↓ ↓ ↓ ↓ | ↓ ↓ ↓ | ↓ |
|  | IL-6 | ↓ ↓ ↓ | ↓ ↓ ↓ ↓ | ↓ ↓ ↓ ↓ | ↑ ↑ |
|  | IL-12p40 | ↓ ↓ ↓ ↓ | ↓ ↓ ↓ ↓ | ↓ ↓ ↓ ↓ | ↓ |
|  | IL-10 | ↑ ↑ ↑ ↑ | ↑ | ↑ ↑ | ↑ |
| **Evaluation of *T. cruzi* Invasion** | *T. cruzi* invasion | ↑ | ↑ | ↑ | N/A |
|  | IL-6 | N/A | N/A | ↓ | N/A |
|  | IL-12p40 | N/A | ↓ | ↓ | N/A |
|  | IL-10 | N/A | N/A | N/A | N/A |
| **Presence of PGE2** | PGE2 | N/D | N/D | N/D | N/D |
